# Making a case for the consideration of trust, justice, and power in conservation relationships

**DOI:** 10.1111/cobi.13903

**Published:** 2022-04-26

**Authors:** Omar Saif, Aidan Keane, Sam Staddon

**Affiliations:** ^1^ School of GeoSciences University of Edinburgh, Institute of Geography Edinburgh UK

**Keywords:** conservation organizations, conservation work, Indigenous people, justice, local communities, political ecology, trustworthiness, comunidades locales, ecología política, fiabilidad, justicia, organizaciones de conservación, pueblos indígenas, trabajo de conservación, 公正, 信誉, 政治生态学, 原住民, 当地社区, 保护工作, 保护组织

## Abstract

In conservation, trust and justice are increasingly recognized as both intrinsically valuable and critical for successful socioecological outcomes. However, the interdependence between these concepts has not been explored. The conservation trust literature provides examples of efforts to build trust between conservationists and local actors; yet, these interventions are often conceived to incentivize local cooperation within dominant paradigms. We argue that when trust building is promoted as a technical fix that does not plan in advance to address power asymmetries in conservation practice, inequities may inadvertently be re‐embedded. Therefore, we conceptualized a framework that joins trust, justice, and power so that critical analyses of conservation partnerships can be more effectively undertaken. We drew on environmental justice theory to better calibrate the trust literature for the historical‐political settings of conservation, especially in the Global South. Justice and trust share strong theoretical links where perceptions of justice shape a willingness to trust, and, equally, trust is a precondition for justice to be perceived. Different forms of trust connect to varied domains of justice and power in different ways, which mediates the outcomes of interventions. We applied our framework to case studies to explore how these interdependences play out in practice. Failure of agencies to attend to issues of maldistribution, misrecognition of cultural values and knowledge, and exclusion from participation strongly compromised trust. Moreover, the ways in which nature‐dependent communities and marginalized conservation workers are trusted, or the conditions under which they give trust, can lead to partnerships being perceived as just or unjust. Focusing on trust and justice can help identify power dynamics so they can be addressed more readily and create space for alternative understandings of partnerships.

## Resumen

La confianza y la justicia cada vez son más reconocidas como valores intrínsecos y de suma importancia para los resultados socioecológicos exitosos en la conservación. Sin embargo, no se ha explorado la interdependencia entre estos conceptos. La literatura sobre la confianza de conservación proporciona ejemplos de esfuerzos por forjar confianza entre los conservacionistas y los actores locales; sin embargo, con frecuencia se concibe que estas intervenciones promueven la cooperación local dentro de paradigmas dominantes. Argumentamos que cuando se fomenta la formación de la confianza como un arreglo técnico que no planea por adelantado abordar las asimetrías de poder dentro de la práctica de la conservación, las desigualdades pueden ser reintroducidas involuntariamente. Por lo tanto, conceptualizamos un marco de trabajo que integra a la confianza, la justicia y el poder para emprender de manera más efectiva los análisis críticos de las colaboraciones de conservación. Partimos de la teoría de justicia ambiental para calibrar de mejor manera la literatura sobre la confianza para el contexto histórico‐político de la conservación, especialmente en el sur global. La justicia y la confianza comparten vínculos teóricos fuertes en donde las percepciones de la justicia dan forma a una voluntad por confiar, y de igual manera, la confianza es una precondición para que la justicia sea percibida. Las diferentes manifestaciones de la confianza se conectan de diferentes maneras con dominios variados de la justicia y el poder, lo que media los resultados de las intervenciones. Aplicamos nuestro marco de trabajo a algunos estudios de caso para explorar cómo estas interdependencias se desarrollan en la práctica. La incapacidad de las agencias para atender temas de mala distribución, los errores en el reconocimiento del conocimiento y los valores culturales y la exclusión en la participación pusieron en peligro a la confianza. Más allá, las maneras en las que se confía en, o las condiciones bajo las que otorgan confianza, las comunidades dependientes de la naturaleza y los trabajadores marginalizados de la conservación puede llevar a que las colaboraciones sean percibidas como justas o injustas. El enfoque sobre la confianza y la justicia puede ayudarnos a identificar las dinámicas de poder para que puedan abordarse más prontamente y crear espacio para el entendimiento alternativo de las colaboraciones.

## 【摘要】

人们正越来越多地认识到保护中信任和公正的内在价值, 及其对成功的社会生态结果的关键作用。然而, 这些概念之间的相互依赖关系尚未得到探索。关于保护信任的文献提供了保护者与当地行动者建立信任的案例;然而, 这些干预措施往往被认为是在主流范式下激励当地合作。我们认为, 当保护者将建立信任作为一种技术性解决方案进行推广, 但却没有事先计划解决保护实践中的权力不对等问题时, 可能会在无意中引入不公正。因此, 我们构建了一个框架来整合信任、公正和权力, 从而更有效地批判性分析保护中的伙伴关系。我们借鉴环境正义理论, 更好地确定了历史政治背景下关于保护信任的文献, 特别是在南半球。公正和信任在理论上有很强的联系, 即对公正的感知构成了信任的意愿, 而信任同样也是感知公正的前提条件。不同形式的信任以不同的方式与公正和权力的各个领域相联系, 影响着干预措施的结果。我们将该框架应用于案例研究, 以探讨这些相互依赖关系在实践中是如何发挥作用的。我们发现, 机构对分配不公问题的忽视、对文化价值及知识的错误认识, 以及对参与行动的排斥, 都严重损害了信任。此外, 依赖自然的社区及边缘化的保护工作者获得信任的方式, 或他们给予信任的条件, 决定了伙伴关系是否被视为公正的。关注信任和公正可以帮助识别权力的动态变化, 从而更好地解决这些问题, 并为对伙伴关系的另类理解创造空间。**【翻译: 胡怡思; 审校: 聂永刚】关键词**

## INTRODUCTION

Trust building is increasingly seen as crucial to forging better conservation relationships necessary to stymie biodiversity decline (Dietsch et al., 2021; Stern & Coleman, 2015). Willingness to accept or resist conservation initiatives is also contingent on whether they are perceived as socially just (Martin, 2017). The Roman philosopher Marcus Tullius Cicero remarked in *De Officiis* (43 BCE) that the foundation of justice is good faith, implying a fundamental interdependence between justice and trust. However, while a wide literature discusses trust and justice separately, a synthesis of why they should be considered together is lacking, which we sought to address.

Conservation‐focused trust research is a rapidly growing literature on securing cooperation as a means to an end for achieving conservation agencies’ objectives. Although not problematic in itself, when achieving cooperation for conservation success is prioritized over engagement with historical and politically charged issues, ambitions to build trust can inadvertently reproduce inequalities. The dispossession of peoples from ancestral territories and militarization of biodiverse areas are, for example, widespread dominant conservation models that have produced a deep distrust in modern environmentalism (Goldman, 2011).

Reconceptualizing trust building in conservation away from a one directional target serving persuit is a considerable challenge. For example, agencies giving trust to communities entails accepting vulnerability and requires relinquishing control and power (Li, 2007) over management, resources, or ideas (Hughes & Vadrot, 2019). However, this seems an unlikely concession because the fundamental premise of Global North conservation has historically and often continues to be based on a mistrust of (usually Global South) rural actors' relations with nature (Li, 2007). This is characterized by fine‐and‐fence approaches and programs to change environmental behaviors of Global South populations (Kashwan et al., 2021).

These legacies of experiencing distrust consequently produce negative attitudes toward conservation and skepticism in well‐meaning conservation practitioners and researchers (Massé, 2020). Indeed, conservationists face intractable challenges in overturning these historically determined relations and pursuing just conservation because they operate within structures (e.g., donor accountability) that constrain their agency (Benson Wahlén, 2014). Our critical appraisal of trust building does not intend to diminish the emotional labor of practitioners who strive for positive relationships. Conversely, we sought to express what many practitioners tacitly know;Thattrust can be gained or lost in explicit relation to perceptions of justice and processes of trusting may shape perceptions of justice.

We suggest now is a particularly amenable time to redefine conservation relationships. There is increased attention on human rights violations, with donor funding retracted due to public outcry, while Indigenous peoples and local communities’ (IPLCs) contributions to biodiversity conservation are increasingly evidenced (Sze et al., 2021). Critics within the conservation community are pushing for changes to dominant conservation models (Díaz et al., 2019) and calling for decolonization of conservation, in the field and workplace, frequently in the pages of mainstream journals (Trisos et al., 2021). A common thread across these criticisms is increased attention to power and active shifts in historically produced relations. Decolonizing conservation, we suggest, also requires alternative conceptualizations of trust.

By merging trust with the concept of perceived justice in our proposed framework, we seek to facilitate a critical analysis of varied conservation relations and to uncover and address power asymmetries. As an intentionally wide‐ranging essay, we prioritized breadth over depth and explore partnerships between conservation actors and the people locally affected by conservation initiatives. We considered the challenges of marginalized conservation workers and smaller organizations in their asymmetrical relationships with larger institutions.

In our review of the conservation trust literature, we looked for insights and overlooked issues and considered political ecology to frame understanding of power. We used environmental justice as a grammar to address the limitations of the trust literature and recalibrate for conservation relations in the Global South. Building on this conceptual scaffolding, we explored the theoretical interdependences between trust and justice through examples drawn from new projects, midterm interventions, and institutionalized conservation programs. We sought to provide an exploratory model for justice and trust in conservation.

### The role of trust in conservation

A focused review of conservation research concerning trust (Appendix S1) provides a background on which we built our central arguments and critique. To maintain linguistic and conceptual precision, we used *trust* to mean the *“*willingness to be vulnerable based upon positive expectations of the intentions or behaviors of another*”* (Rousseau et al., 1998). Trust is a leap of faith, whereby individuals (trustors) believe that the trustee (an individual, an institution, or set of rules) will act favorably on their behalf and encapsulate their needs (Stern & Coleman, 2015). Trustworthiness pertains to individual or institutional benevolence, integrity, and ability (Colquitt & Rodell, 2011). Lack of trust and mistrust suggest ambivalence, whereas distrust suggests a relationship has been compromised (Stern & Coleman, 2015).

Studies often explored how to build trust under assumptions that trust was a scarce social resource. In cases of community discontent, studies explored barriers, such as lack of trustworthiness of protected area managers (Stern, 2008), scientists, or science (Shirley & Gore, 2019). Most concluded that trust is fundamental for effective management and necessary for conservation success (Stern, 2008; Hamm et al., 2016), whereas distrust is dysfunctional, hindering parties from engaging in conflict resolution (Young et al., 2016). However, not all studies simply advocated for more trust. Research also highlighted the functional value of distrust. In the context of community‐based resource management, small doses of distrust can be constructive when skepticism of elite actors’ self‐interested motives elicits wider participation in decision‐making processes (Idrissou et al., 2013).

From a theoretical perspective, studies split trust into different forms, reflecting developments in trust scholarship (Lewicki et al., 2005). Stern and Coleman (2015) argue that “typologies” of trust can reveal where one form may be scarce and where trust repair is needed (Table 1). We considered how different forms of trust are useful for identifying perceptions of justice and power asymmetries in conservation relationships.

**TABLE 1:** The typologies of trust from Stern and Coleman (2015) contextualized with hypothetical conservation examples representing both community and conservation agency perspectives

Form of trust	Definition	Examples in conservation
Confidence based	A rational economic perspective based on the evaluation of past performances, predictability, and consideration of costs and benefits of a relationship. Trust here relies on information the trustor can gather on the trustee to make a calculated assessment.	A community receives prompt and adequate compensation for wildlife damage, therefore, building confidence in the agency.
		An agency lacks confidence in a community's regulation of resource use, and instead employs the use of drones to monitor and enforce activities.
Dispositional	An actor's predetermined affinity to trust that can be context dependent or independent (e.g., propensity to trust those with a certain title, or the tendency to distrust governments, institutions, or objects based on their perceived legitimacy and authority).	A community's predisposition to be suspicious of foreign researchers arriving with maps of local territories.
		An agency's lack of trust in certain marginalized rural actors, such as fishing and hunting groups based on an assumption that they excessively exploit resources compared with others.
Affinitive	Shaped by shared values, identities, and feelings of social connectedness; consciously developed through evaluation of character or subconsciously through automatic responses of trustees’ personality or charisma leading to shared or differing values.	Villagers’ affinity with conservationists who empathize with their concerns.
		An agency that develops trust in indigenous actors upon understanding the richness and depth of their ecological knowledge when it echoes their own scientific values and principles.
System‐based (also termed as procedural trust)	Concerning fair procedures and practices (i.e., when the system is agreed upon as fair by all actors involved, there is greater confidence in the compliance of others).	A community develops trust through an agency's diligent practice of free, prior and, informed consent and early‐stage consultations on the scope of an intervention.
		Conservation workers lose trust in their organization as avenues to give critical feedback on project implementation are censored.

### Problematizing the lack of attention to justice and power

Our inductive reading of the trust literature revealed justice and power as salient concepts; broad references were made to fairness, legitimacy, and justice. However, despite the inclusion of these concepts, their articulation lacked detailed explanation and did not reference substantive theory (Appendix S1). Understandings of justice were limited to fair procedures or material distribution, avoiding more radical notions of justice, and failed to appreciate how dimensions of justice are contingent on each other (Fraser, 2008). Moreover, studies did not link the mechanisms by which trust is affected by perceptions of justice and vice versa.

Prevalent framings were how to build trust to ensure compliance, remove local opposition, and encourage belief in conservation. Hamm et al. (2016) conclude, *“*natural resource management institutions would likely be most efficient in increasing cooperation if they directly address stakeholders’ willingness to be vulnerable to them,” whereas others suggest that trust in scientists would “result in more favorable conservation outcomes because of more consistent and widespread compliance with environmental rules” (Shirley & Gore, 2019). These instrumental framings understandably emphasize the potential practical benefits of building trust. However, they also reproduce “narratives that maintain an organization's definition of the problem” (Mosse, 2004). In many cases, trust has been “rendered technical” (Li, 2007); expert‐derived prescriptions are presented to solve the problem of distrust without disturbing the political status quo that may be perpetuating distrust in the first place.

The literature strongly emphasizes incentivizing communities to trust conservation agencies, suggesting deep unidirectionality. Although this may not necessarily reflect the nature of relationships on the ground, it represents a lack of interest in how conservation agencies could better trust local actors and their practices, thereby disrupting dominant positions of trustor and trustee. Too often, trust building is misleadingly framed as equal parties coming together to fulfill an assumed shared purpose, but this favors conservationists’ concerns while ignoring their potential direct contention with locally defined notions of justice.

The subject of power in relation to trust and trustworthiness, although encouragingly mentioned in the literature, is a topic we found lacked in‐depth exploration (although see Dietsch et al. [2021]). This was also found to be a shortcoming in the wider trust literature (Möllering, 2019). Powerful discourses (produced through scholarship or media) can negatively shape perspectives of other cultures, predisposing willingness to trust certain actors and not others (Said, 1978). Particularly for the typically donor‐driven conservation sector, power determines to whom agencies are accountable (Jepson, 2005) and, therefore, whose trust they need to win. Power, we will show, is indispensable in analyzing trust. Questions of who has the power to build trust and the potential consequences thereof remain largely unexplored in conservation (Horowitz, 2010), and we explored them through a political ecology lens.

Political ecology is a field that foregrounds power (Robbins, 2012). It questions who controls the language and influence (i.e., discourse) that, for example, lead many people to believe the Serengeti is a wilderness, historically devoid of humans, that is now being encroached upon by the rural poor (Robbins, 2012). These constructions have material impacts through their influence on the framing and design of conservation interventions. Conceptually, power takes three forms (Svarstad et al., 2018): actor‐based power, structural power, and poststructural power (Table 2). In practice, they often operate in tandem.

**TABLE 2:** Definitions and conservation examples of the forms of power related to conservation based on theory and empirical examples from political ecology

Form of power	Explanation	Example in conservation
Actor based	Power exercised by or through actors to realize that actor's will, despite resistance from others. Power resources are the various types of capital people can use to realize their intentions and can be material (e.g., wealth), ideational (e.g., influential narratives), relate to capacity (e.g., knowledge), or relational (e.g., networks) and are possessed by actors or activated when needed (Svarstad et al., 2018). Power is not ingrained in a person but changes according to multiple factors (e.g., Academicians are powerful when teaching a class but their power diminishes when they leave campus.).	A U.S. delegation rejected the term *biocultural* heritage being incorporated into policy text in IPBES's^a^ inaugural *Thematic Assessment on Pollinators, Pollination and Food Production*, for fear that such an alternative framing of human nature relations could lead to, for example, political action for positions they oppose. Actor‐based power and thus influence was harnessed by drawing on the powerful legitimacy of scientific knowledge (as an ideational capital asset) to dispute the validity of the term *biocultural*, resulting in its exclusion from the agreement despite Global South country delegates supporting its inclusion (Hughes & Vadrot, 2019).
Structural power (neo‐Marxist perspective)	Structures include political‐economic systems, such as capitalism and colonialism. Structures shape the extent to which power can be exerted and the limits agents are constrained by. Structures condition the agency of individuals and are enacted through centers of power from the local to the centers of national metropoles (Robbins, 2012).	A middle age Mozambican ranger on separate occasions waited in an ambush for rhinoceros poachers, and twice, did not shoot them. He seemingly, through his agency, overcame structural institutional pressures to enforce lethal methods. However, he could not transcend broader structures of power. He was formally reprimanded for his actions. Further, he was socially rebuked and marginalized by colleagues who were strongly conditioned to support the paramilitary conservation paradigm. Despite dissenting actions and even because of them, normalization and social acceptance of using lethal force to secure conservation territory remains intact, unchallenged, and reaffirmed (Massé, 2020).
Poststructural power	Discursive power is the ability to establish and disseminate discourses on issues and narratives that others adopt and reproduce in ways that are suitable to one's own strategic interests. Governmentality in its disciplining form implies the ways in which people come to self‐govern, such that their interests and ways of being become aligned with dominant societal structures and powerful institutions and forces (e.g., the state or capitalism). People come to internalize these social norms and ethical standards as their own (Svarstad et al., 2018).	For example, appropriation by international nongovernmental organizations and states of new conservation territories in the Global South is facilitated through the creation of narratives that local inhabitants use resources in an unsustainable manner. Environmentality implies a process in which the influence of environmental governance institutions leads to the creation of environmental subjects (i.e., conservation‐minded people with new identities and values that differ from before interactions with environmental agencies (Svarstad et al., 2018).

^a^ The Intergovernmental Science‐Policy Platform on Biodiversity and Ecosystem Services.

Different forms of power shape trust in complex ways. A conservation agency's unwillingness to trust in IPLC's ecological knowledge and values (an affinitive mistrust) implies a reluctance to give up the power of scientific authority (agential and discursive power) and, by extension, legitimacy for implementing interventions (Hughes & Vadrot, 2019). Forms of power also shape the connections between justice and trust, which we make explicit here to force consideration of the political realities and asymmetries inherent in trust building.

### Environmental justice as an appropriate grammar for conceptualizing trust

To move beyond trust building as a technical intervention that masks vested interests, one must explore which factors shape distrust, perpetuate power asymmetries, and produce feelings of injustice. We used environmental justice as a key framework to undertake this. This scholarship and adjoining activism originated from civil rights struggles in the United States in reaction to the unequal distribution of environmental pollution, such as chemical dumping in areas inhabited by people of color (Schlosberg, 2004). Exposure to environmental risks generally correlated with “inequity in socio‐economic and cultural status” (Schlosberg, 2004). We followed the tripartite definition comprising interacting dimensions of distribution, recognition, and representation (Fraser, 2008), increasingly adopted in conservation contexts (Martin, 2017; Strzelecka et al., 2021). We suggest a contextual understanding of justice because justice is perceived variably by different actors according to particular situations, values, and identities (Martin, 2017), as opposed to relying on universal theories to define what is just.

#### Distribution justice

In conservation, distributional injustices include the high opportunity costs IPLCs face in coexisting with wildlife, in their unequal share of the benefits of wildlife tourism, or in their eviction from protected areas; achieving international biodiversity goals are argued to accrue at the expense of the poor (Martin, 2017). The dominant framing of justice in the conservation trust literature follows ideals from traditional liberal moral philosophy (Schlosberg, 2004), which focuses on procedures leading to equitable distribution of benefits.

However, feminist social theory, which informs environmental justice, upholds that, whereas *“*theories of distributive justice offer models and procedures by which distribution may be improved, none of them thoroughly examine the social, cultural, symbolic, and institutional conditions underlying poor distributions in the first place” (Schlosberg, 2004). Environmental justice illustrates how interventions based only on improving distribution are detached from their underlying cause and can assimilate target communities into dominant ways of relating to nature. For example, ecotourism is premised on equitable distribution of revenue with local inhabitants of wildlife areas, but imposes a world view where nature is only of value when it is financialized, displacing other ways of connecting with and stewarding land and seascapes (Martin, 2017).

#### Recognition justice

Central to distributional justice is, thus, recognition justice. This refers to, at minimum, the respect of diverse values, knowledges, genders, ethnicities, castes, classes, and abilities. It further entails the recognition of unequal power structures within norms in society and avoidance of cultural and cognitive domination (Martin, 2017). Many recognition injustices have their genesis in racism and imperialism, which may be maintained despite material wealth (Fraser, 2008). Misrecognition legacies persist through racially differentiated conservation enforcement policies. For example, large‐bodied mammals are critical in maintaining intact ecosystems; however, legally mandated shoot‐to‐kill policies to stop hunters are only implemented in Global South countries and not in North America (Kashwan et al., 2021).

Another key aspect of recognition is respect for diverse knowledge forms (epistemic justice). Epistemic injustices occur when the use of certain criteria (dominant scientific principles) and their associated logics are used to judge other epistemologies (e.g., local ecological knowledge). This demarcates privileged groups whose knowledge is respected from those excluded. Taking it a step further, epistemic oppression involves powerful actors censoring other knowledges or experiences from being understood by wider society, resulting in epistemic exclusion that inhibits these actors’ abilities to contribute and participate in decisions and influence social understanding (Fricker, 2007).

#### Representation justice

Representational (or procedural) justice is inherently about struggles over political membership and being recognized as eligible to participate. It delimits who is entitled to make justice claims (Fraser, 2008). A major form of misrepresentation occurs when political structures prevent fair participation, such as holding comanagement decision meetings during harvest time, preventing equal participation.

Misrepresentation occurs more seriously as misframing, or an active exclusion of the ability to participate. Global South actors seeking redress for injustices must typically pursue their claims within nation states, curtailing their ability to challenge transnational organizations which cause their marginalization (Fraser, 2008). This is of particular concern with international nongovernmental organizations (INGOs) (Rubis & Theriault, 2019). In Madagascar, an INGO directly influenced the government to expand protected area networks to the detriment of local actors, who were left to contest this injustice with unconcerned government officials. Holding the INGO accountable was impossible because they were outside the local communities’ reachable political boundaries (Duffy, 2006).

### Unpacking the relationship between environmental justice and trust

Concepts of distribution, recognition, and representation justice are important for understanding trust dynamics in conservation, particularly in the Global South. They are linked in multiple ways that we considered through case studies. To help structure our synthesis, we first outlined the mechanisms through which trust, trustworthiness, and justice can be connected.

First, justice can be a precondition for trust and trustworthiness (Lewicki et al., 2005).

Trustors’ assessments of whether their interests will be encapsulated and their decisions to trust can be shaped by perceptions of the trustee's justice values and related actions (Colquitt & Rodell, 2011). Experiences of being at the receiving end of discriminatory policy (i.e., injustice) can drive feelings of vulnerability, reducing a willingness to trust. In addition, violations that compromise a trustor's notions of justice reduce the trustor's perception of the trustee's trustworthiness. Conversely, fair treatment engenders reciprocation and a willingness to trust.

Second, trust and trustworthiness can be preconditions for perceptions of justice. For example, being trusted provides an individual with an affirmation of self‐worth and recognition of their identity (Lewicki et al., 2005). Conversely, an actor may bear costs to maintain the trust of a more powerful trustee, creating a perceived injustice (Graham, 2017). Doubting an actor's trustworthiness may also produce feelings of justice or injustice (Rawls & Duck, 2017).

The three dimensions of justice variably act as preconditions for gaining or losing a sense of trust and trustworthiness, moving from distribution to recognition to representation. And, trust and trustworthiness can be preconditions for perceptions of justice.

#### Perceived justice as antecedent to trust and trustworthiness

**FIGURE 1:**
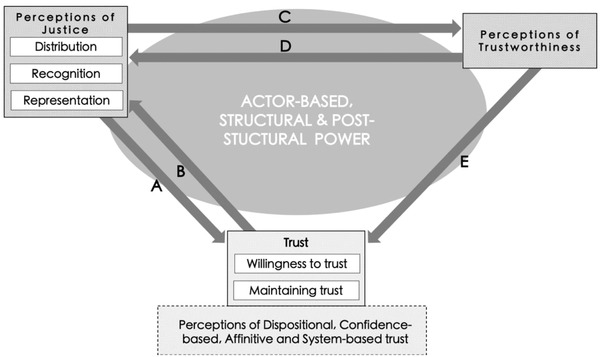
Framework of conservation trust and justice based on trust, environmental justice, and political ecology scholarship. Letter labels on arrows correspond to case studies described in text and show relationships between components of the framework These connections are shaped and mediated by actor‐based, structural, and poststructural power

Distributional justice is perhaps the best known dimension of justice that shapes trust in conservation relationships. For example, when wildlife damages occur, conservation agencies that implement well‐distributed compensation build a confidence‐based trust with communities (Ravenelle & Nyhus, 2017). Conversely, distrust emerges when promises are not upheld, such as benefits from tourism not materializing or capacity‐building workshops constituting large opportunity costs for IPLCs. These shape perceptions of injustice that result in skepticism toward conservation (West & Aini, 2021). Distributive concerns and their impact on trust are often linked to broader issues of recognition. When wildlife managers prioritize habitat protection for biodiversity and restrict human access, misrecognition manifests by undermining locally defined understandings of reciprocity and human–nature relationships. Material burdens that stem from or are understood to occur because of differences in value systems may result in distrust of western conservation values (joint maldistribution and misrecognition produce affinitive distrust). Such interdependences are represented by connection A in Figure 1.

Misrecognition strongly affects perceptions of trustworthiness, particularly in racialized settings. In sub‐Saharan conservation workplaces, misrecognition of personhood through the construct of race led to assumptions that Black conservationists are more prone to corruption than their White colleagues and, therefore, cannot be trusted with finance‐based or higher managerial positions (Duff, 2020). Legacies of colonialism and orientalism produced barriers in seeing marginalized actors' outside racialized lenses, reducing their agency and identities to a generalized category (Said, 1978), wherein they are assumed to be untrustworthy.

Similarly, being socially categorized as coming from a privileged background implies misrecognition and a similar challenge of an actor's trustworthiness. Massé (2020) recounted his experience in Mozambique: “People do not trust a white foreigner poking around asking questions about poaching, and for good reason. Being white, many people believed I worked for the 'Park,' no matter how hard I, my research assistants, and local friends tried to demonstrate otherwise.” The social identities one recognizes as having social legitimacy and can trust (and those one does not extend this privilege to) are ingrained in the historical events, power dynamics, and politics that shape assumed trustworthiness. These examples characterize connection C in Figure 1.

Such biases also extend to the trustworthiness of certain actors’ knowledge. Epistemic injustices, which occur when traditional and Indigenous knowledges and their custodians are disregarded, illustrate how misrepresentation and misrecognition drives distrust. In a study with a conservation NGO and artisanal fishers in the Seychelles, where the former engaged the latter in a citizen science project, the scientists were unwilling to include the fishers until scientific tests verified that local knowledge could accurately provide the fish population data needed (Baker & Constant, 2020). Doubting the trustworthiness of their knowledge emerged from structurally formed dispositions in what counts as science and expertise. Moreover, only a limited spectrum of the fishers’ knowledge (taxonomic identification) was used to support marine protected area prioritization, while their knowledge on shifting fish breeding grounds gathered from decades of experience was ignored. Primacy of dominant science validated certain forms of knowledge as amenable to project goals while excluding others, rendering it and their producers unknown to society and outside the remit of political decision‐making (Fraser, 2008). This made apparent that conservation would not encapsulate marginalized communities’ interests because their voices and knowledge were not recognized, thereby producing distrust (Baker & Constant, 2020).

These cases show how perceptions of justice shaped perceptions of trustworthiness or willingness to trust. Table 3 contains further examples and explicitly outlines the forms of justice, trust, and power involved. Trust and trustworthiness in turn can affect perceptions of justice and power can mediate this relationship.

**TABLE 3:** Summary of potential connections between perceived justice and trust and trustworthiness and outcomes of perceptions of justice and influences of the multiple forms of power

Perceived justice as precondition	Example	Role of power	Trust outcomes	References
Perceptions of intergenerational maldistribution and contemporary misrecognition	By not recognizing the impacts of former colonial policies in Tanzania that displaced a particular Maasai community, conservation organizations seeking to prohibit the same tribal‐lineage of pastoralists from their grazing practices further exacerbated a long‐standing distrust in conservation	Structural power of colonial institutions that permit conservation's ability to operate and maintain control in this Global South location	Reproduction of long‐standing confidence‐based distrust System‐based distrust in conservations institutions and models of operating	Goldman, 2011
Perceptions of misrecognition and misrepresentation	Initial exclusion of farmers from Natura 2000 site designation led them to perceive that their environmental heritage was ignored. Despite later efforts to introduce participatory mechanisms, many stakeholders remained distrustful and were skeptical of these latter attempts to include them in management planning	Structural inequality in which the Polish environmental regional units had limited power and resources to secure initial funding needed for participatory consultations	Lack of affinity and trust in Natura 2000 decision makers because local values were not considered wider system‐based distrust in general conservation practice	Strzelecka et al., 2021
Perceptions of recognition and participation	In public meetings on local energy disputes, it was simple perceptions of recognition and basic representation justice facilitated by actions of local government that led to trust. For example, people could easily participate (e.g., parents with children could speak first so they could go home early or speech time limits were ignored so attendees could really express their concerns)	Actor‐based power of local government agents created a safe political space to share concerns.	Recognition of individuals everyday needs and provision for meaningful participation led to a strong affinity with local council actors and trust in local government. One respondent related; “I trust the judgment of you all. I know almost all of you all, and I ultimately trust your judgment to do what's right for us. I got to express my concern earlier”	Marlin‐Tackie et al., 2020

Trust as precondition	Example	Role of power	Outcome on perceptions of justice	Reference
Trust given to secure justice	In New Caledonia, trust was given to a mining institution based on their affiliation with its employees and predictions of what the relationship may provide in the future. For some parts of a community, this was premised on long‐term expectations that projects would allow people to live better, suggesting perception of a forthcoming distributive justice was justification enough to trust. For others, affiliation depended on anticipation that siding with an institution would provide them with more political autonomy	Expectations that the agency planned to remain for the long term provided some members of the community with a sense of their actor‐based power over it. This was due to perceptions that if the agency wanted to remain in the long run, it was in their interest to give in to community needs	Trust leads to perceptions that distributional and representation justice will be forthcoming	Horowitz, 2010
Performance of trustworthiness	In Vietnam, government‐led media campaigns widely advertised the introduction of payment for ecosystem services (PES) schemes as an ecological and social success to justify its roll out nationwide. This selling of success propped up failing forest institutions, allowing them to perpetuate injustices as claimed by local communities	Power is enacted in multiple ways. State officials and donors, through actor‐based power and material resources, influence a web of contacts to increase support for PES narratives. This allows coherence in selling success, which is a form of discursive power	Process invalidates marginalized actors’ claims to justice because their claims are overshadowed by pervasive narratives of success. This also produces feelings of misrecognition and being ignored, leading to a epistemic injustice (e.g., their voices are made unintelligible to wider society) and lack of representation and participation in debating environmental policy	To and Dressler, 2019
Maintaining trust of more powerful institutions	Ailan Awareness (AA), a small NGO from Papua New Guinea, endures substantial costs to maintain a relationship it has with an INGO that it partly derives its funding from. Through their connections to Global North universities, the INGOs often recommend students conduct research with AA. Often unprepared and socially unaware of the culture they arrive in, students arrive demanding logistic and research support that AA feels it must reciprocate. In these exchanges, AA staff accrue distributional costs and social debt and draw on kinship networks to grant the access the student needs. Later, AA will need to reciprocate long after the student is gone. These students or research teams often act in privileged and entitled ways that produce harm to hosts through insensitivity to identity, ethnicity, and class. Moreover, perceptions of disrespect emerge in this exchange when outsiders are not satisfied with these arrangements	Poor structural position of AA staff leads them to accept the costs of this partnership and undertake invisible and socially degrading work. Although not officially mandated to, small NGOs must bear the costs in fulfilling this unspoken accountability to remain trusted and funded	Indigenous NGO hosts perceive high distributional costs, such as financial, time‐based, and emotional deficits. Feelings of misrecognition and disrespect result from the exchange. The NGO staff are not observed in terms of parity and rather expected to fulfill the role of service provider	West and Aini, 2021

#### Trust and trustworthiness as antecedents to perceptions of justice

Being willing to trust, maintaining trust, and performing as trustworthy each have important justice and power‐related implications. In New Caledonia, the Kanak Indigenous activist group fighting industrial sea mining in the world's second largest barrier reef believed the reef's designation as a UNESCO world heritage site could help save it. UNESCO was perceived as a powerful environmental institution that would be aligned with their anti‐industrial grassroots activism and able to leverage influence. The Kanak thus applied and succeeded in gaining UNESCO world heritage status, trusting that it would encapsulate the Kanaks’ needs as their own. However, this trust was misplaced because through nested power relations, UNESCO was beholden to the New Caledonian and French government's interests, which supported mining operations. By partnering and trusting in UNESCO, the Kanak forwent their ability to actively oppose the mine because they were enfolded into both UNESCO's diplomatic inaction and a community‐based project to reduce their artisanal fishing practices. Furthermore, the partnership required the Kanak to respect French legal code, which reduced their ability to participate politically and represent their interests in addressing the mine's effluent (Horowitz, 2016).

The relationship between the Kanaks and UNESCO is not atypical of conservation relationships. We used this example to illustrate how trust was mediated by power.

First, UNESCO's high actor‐based power engendered the Kanaks’ willingness to trust, through a hope of affiliation with power and gaining influence (Horowitz, 2010). By choosing to maintain that trust, the Kanaks had to forgo their resistance, limiting their actor‐based power. Second, from the perspective of UNESCO staff, trust could not be reciprocated due to structural power dynamics. Human agency is often constrained by social structures and people do not make decisions “under self‐selected circumstances, but under circumstances existing already, given and transmitted from the past” (Marx [1852] as cited in Svarstad et al. [2018]). Here, the accountability structures inherent in the global politics of corporate and state finance limited UNESCO personnel's ability and power to side with the Kanak and challenge the capitalist interests of the French state and mining industry. Actor‐based and structural power thus influence willingness to trust and the ability to reciprocate that trust, highlighting how power mediates partnerships that result in perverse environmental outcomes and perceptions of injustice.

Structural power‐related vulnerabilities, such as economic poverty and employment needs, also reduce the ability for IPLCs to withhold trust. In rural conservation areas in the Global South, precariously contracted conservation workers are commonly employed to regulate community resource use. Hired by state agencies and NGOs at low wages, these agents are expected to be exemplar environmental representatives. Yet, maintaining the agency's trust can necessitate forgoing important aspects of one's cultural heritage, livelihood activities, and social relations (Haenn, 2016). In relating challenges of forest workers, Dutta showed how they are pressured to “prove their loyalty to the forest department” (i.e., gaining or maintaining trust) by arresting members of their own community (Dutta, 2020). This can lead people to “adopt identities distinct” (Haenn, 2016) from the communities they belong to and become alienated.

Trust of this character is, therefore, highly contingent on being a good environmental subject. Even without monitoring, being conditionally trusted by a powerful actor or organization often leads to the adoption of self‐regulating behavioral changes, termed environmentality (Robbins, 2012). This is a concept of poststructural power in which actors come to self‐govern and display values reflecting interests of powerful institutions. Only then are these rural contractors perceived as rational (and we add trustworthy) environmental subjects (Rubis & Theriault, 2019). In many cases, IPLCs and rural workers “cannot be trusted automatically to do the right thing. Therefore, they need to be tutored, their conduct conducted in appropriate ways” (Li, 2007). This is reminiscent of colonial paternalistic legacies that remain a challenge to dismantle. Being trusted and maintaining such conditional trust can thus drive feelings of misrecognition (e.g., that their current environmental values are of little worth) and compromises the ability to represent oneself in society and be socially included. These interdependences represent connection B in Figure 1.

In other instances, to continue being seen as trustworthy professionals, marginalized actors internalize injustices resulting from structural power relations. As a Black South African conservation manager reflected: “One time I was racially discriminated and stuff in the workplace by a subordinate. It is difficult, [if I speak out] then that person maybe might lose his job and then I'll be responsible, so let me rather be silent [about] that.” (Graham, 2017). Unlike in earlier examples, where historical structures led to misrecognition and consequently a doubt of trustworthiness; here, the manager accepted misrecognition and disrespect of their personhood to maintain an outwardly cordial and problem‐free working environment and retain the trust of the employer, a silenced cost that is rarely acknowledged (Rawls & Duck, 2017). This relation may also manifest between local NGOs and INGOs (Table 3) (West & Aini, 2021). This characterizes connection D in Figure 1.

In contrast, powerful actors, such as INGOs, may wield trustworthiness as an asset. There is a strong incentive to do this because having power without being trusted reduces one's ability to influence (Möllering, 2019). Without appearing trustworthy to their donors and the public, INGOs would not be able to legitimately operate (Jepson, 2005). Agencies thus need to exhibit trustworthiness and organizational effectiveness to those they are accountable to, which can result in practicing selective ignorance regarding conservation's negative outcomes (Benson Wahlén, 2014) or “selling success” (To & Dressler, 2019). Taking it a step further, INGOs may utilize their public relations departments to recast conservation human rights abuses in a positive light, actively shaping an image of trustworthiness (Domínguez & Luoma, 2020). The use (or abuse) of discursive power by institutions to produce favorable narratives and appear trustworthy renders marginalized voices unintelligible, unheard, and unrecognized by a wider public (To & Dressler, 2019; Baker & Constant, 2020), reducing their political space for representing their concerns (Fraser, 2008). Recognizing the heterogeneity of local communities, power differences, such as along lines of caste, gender, and class, can also determine who is perceived as trustworthy. For example, elite groups tend to perform the behavior‐value orientations conservation interventions intend to achieve and desire, incentivizing agencies to trust them and prioritize working with them to ensure project success (Li, 2007). Trust as performance can thus obscure marginalized actors’ abilities to be claim justice. Perceptions of untrustworthiness lead to unwillingness to trust in conservation, through a lack of confidence or skepticism in the overall system or values it is underpinned by (confidence‐based, system‐based, and affinitive distrust) (connection E in Figure 1).

Conservation organizations and state agencies are themselves embedded in wider geopolitical power structures. Their need to derive legitimacy and appear trustworthy is achieved by fulfilling economic demands or generating value for more powerful actors, such as their donors (e.g., corporations or governments). However, what is often lost is a normative accountability derived from working for social benefit instead of profit and encapsulating the concerns of the people where they operate (Jepson, 2005). Gupta (2014) argues this also occurs to local NGOs that initially gain trust and legitimacy by challenging injustices on behalf of IPLCs, but subsequent expansions and embedding within official development machinery result in their values becoming distanced from their beneficiaries.

Larger institutions are often susceptible to shifts in global political priorities, leading to changes in practices so they can ally with powerful entities to obtain funding. Verweijen and Marijnen (2018) contain an account of paramilitarized conservation being presented as conservation's contribution to the so‐called war on terror. Such organizational policy shifts, however, produce distrust in local communities because communities recognize their needs will not be encapsulated (Gupta, 2014). It is also important to acknowledge that trust given by Global North donors to INGOs because of the discourses they espouse can become a questionable source of legitimacy with far‐reaching implications on local claims to justice in the Global South.

## CONCLUSION

It is necessary to explore the links between justice and trust in the context of conservation practice because the conservation trust literature, despite being a burgeoning field, often advocates trust building uncritically to improve conservation effectiveness. Although well‐intentioned and presented as a neutral solution to resolve conflict, trust building thus acts as a mechanism for gaining IPLC's cooperation within dominant conservation models, thereby neglecting local perceptions of justice. Prior to intervention, donors and agencies may fail to entertain the possibility that free prior informed consent may not be given; trust and partnerships are too often assumed as a fait accompli. Further, without explicitly engaging with power asymmetries in proposed partnerships or workplace relations, structural inequalities are perpetuated. We, therefore, propose an ethics of trust that challenges façades of political neutrality (Abdelnour & Abu Moghli, 2021) and recognizes the inequitable political‐historical‐colonial backdrop in which partnerships are proposed.

Our justice‐trust model can be used to identify and address power asymmetries in conservation relationships, which is particularly important if collaborations with IPLC‐led conservation models are to gain ground. Gaining the trust of historically marginalized actors necessitates not only a justice framework, power‐mapping processes, and context sensitivity, but also likely involves conservation organizations explicitly ceding power and relinquishing control so others may lead. Trust‐building activities, we fear, will continue perpetuating perceptions of injustice as long as they continue to be instrumental and center the rural poor as targets of conservation interventions (Büscher & Fletcher, 2020), as opposed to allies in a partnership seeking to challenge structural drivers of decline. As recounted by an Aboriginal Australian collective: “If you have come to help me, you are wasting your time. If you have come because your liberation is bound up with mine, then let us work together” (Toporek, 2013). Solidarity and a mutual trust with IPLCs may more effectively emerge when conservation organizations are perceived to target colonial‐capitalist structures and their concomitant socioecological injustices.

New conservation approaches and practices can help overcome trust relations that carry colonial legacies of control and conditionalities of partnerships. A concrete example would be a conservation basic income (CBI) (Büscher & Fletcher, 2020), in which unconditional financial support is made to people living in biodiverse areas. A CBI centers distributive justice while recognizing IPLCs’ contributions to nature and their full autonomy. It can be framed as reparations to assuage colonial legacies. Although it does not erase past wrongs, it is an offer to repair relations. Critically, such models switch the dominant trust logics that we have critiqued, asking conservation to trust in IPLC's abilities, knowledges, and worldviews, therfore ceding power and control. This begins to suggest a decolonizing conservation trajectory that goes beyond social justice (Tuck & Yang, 2012). We invite readers engage in alternative possibilities, and create noncolonial forms of partnership.

## Supporting information

Additional supporting information may be found in the online version of the article at the publisher's website.
