# Supplementary material for: Making a case for the consideration of trust, justice, and power in conservation relationships
Source: Conserv Biol. 2022 Apr 26;36(4):e13903. doi: 10.1111/cobi.13903 (PMC9545749; doi:10.1111/cobi.13903)
Supplement: Supplementary file 1 — Additional supporting information may be found in the online version of the article at the publisher's website. [file COBI-36-0-s001.docx]

**Supporting Information for:**

Making a case for the consideration of trust, justice, and power in conservation

**Appendix S1.**

List of reviewed literature on trust and conservation comparing their inclusion of different concepts. Listed by date of publication.

|  | **Reference** | **Study location** | **Directionality of giving trust**  **(community -> agency, agency -> community or bi-directional)** | **Analysis of Power** | **Notions on Justice** | **Article Title** |  |
| --- | --- | --- | --- | --- | --- | --- | --- |
| 1 | (Cvetkovich & Nakayachi 2007) | US | **bi-directional**  Trust levels of four groups in watershed policy, comprising a research institute, two activist groups and the county council | No | Justice as fairness and predicted as a driver of trust.  Perceptions of justice based on our values – suggesting existence of the competing notions of justice | Trust in a High‐concern Risk Controversy: A Comparison of Three Concepts |  |
| 2 | (Davenport et al. 2007) | US | **community -> agency**  Community actors trust in forestry service, agency personnel and management outcomes | Lower power of community leads to perceptions that agencies will compromise trust. | Good procedures may not always gather local support due to historical contexts | Building trust in natural resource management within local communities: A case study of the Midewin National Tallgrass Prairie |  |
| 3 | (Stern 2008b) | US, US Virgin Islands, Ecuador | **community -> agency**  Community to agency trust advocated to assuage active opposition toward neighbouring protected areas | Low power of communities is acknowledged | Fairness of park rangers a strong predictor of the park authority’s trustworthiness | The power of trust: Toward a theory of local opposition to neighbouring protected areas |  |
| 4 | (Leahy & Anderson 2008) | US | **community -> agency**  Community trust in watershed management NRM agency | Limited community power and that power should be shared so that public can influence procedures | Mentioned with regard to procedural justice and equity leading to organisational trust | Trust factors in community-water resource management agency relationships |  |
| 5 | (Stern 2008a) | US, Virgin Islands, Ecuador | **community -> agency**  Community trustworthiness in protected area managers | Measurement of community’s power can help influence decision making | Justice referred to through legitimacy, procedure and distribution. | Coercion, voluntary compliance and protest: The role of trust and legitimacy in combating local opposition to protected areas |  |
| 6 | (Baral & Stern 2010) | Nepal | **community -> agency**  Community members trust in community-based resource managers and committees | No | Legitimacy of authorities required for voluntary compliance | Looking back and looking ahead: local empowerment and governance in the Annapurna Conservation Area, Nepal |  |
| 7 | (Gray et al. 2012) | US | **community -> agency**  Stakeholder trust in the State Fisheries and Wildlife Departments, the Regional Fisheries Management Councils, and the National marine fisheries service and in recreational and commercial science | No | Procedural justice referred to in the introduction. | Understanding Factors That Influence Stakeholder Trust of Natural Resource Science and Institutions |  |
| 8 | (Smith et al. 2013) | US | **community -> agency**  Community trust towards NRM agency | No | No | Community/Agency Trust and Public Involvement in Resource Planning |  |
| 9 | (Sharp et al. 2013) | Australia | **community -> agency**  Community towards researchers and agency | How agencies interact with communities in their exercising their power is important for how trust is built | No | Trust and trustworthiness: Conceptual distinctions and their implications for natural resources management |  |
| 10 | (Idrissou et al. 2013) | Benin | **community -> agency**  Community trust in park management | No | limited benefits to community caused distrust | Trust and hidden conflict in participatory natural resources management: The case of the Pendjari national park (PNP) in Benin |  |
| 11 | (Sponarski et al. 2014) | Canada | **community -> agency**  Rural residents trust in wildlife agency | No | No | Salient values, social trust, and attitudes toward wolf management in south-western Alberta, Canada |  |
| 12 | (Ford et al. 2014) | Australia | **community -> agency**  Community trust in forest landscape management | No | General unjust outcomes of environmental management lead to distrust | Beauty, Belief, and Trust |  |
| 13 | (de Vries et al. 2015) | South Africa | **bi-directional**  Conservationists trust in farmers and farmers trust in conservationists | Discussions of reluctance in handing over power to opposing party | No | Trust related dynamics in contested land use |  |
| 14 | (Metcalf et al. 2015) | US | **community -> agency**  From community to agency and intra-community trust | Lack of power linked to mistrust | Mentions procedural justice | The role of trust in restoration success: Public engagement and temporal and spatial scale in a complex social-ecological system |  |
| 15 | (Stern & Coleman 2015) | Theoretical | **agency -> agency, community -> agency**  NGO staff (dis)trust in industry personnel and NRM managers can increase participants trust in procedures. | Ample discussion of power in different contexts e.g. the power of trustors varies in different situations, and risk of vulnerability not shared equally. | No | The Multidimensionality of Trust: Applications in Collaborative Natural Resource Management |  |
| 16 | (Stern & Baird 2015) | Theoretical | **community -> agency** | System-based trust can be lost, when inequitable management relations exists across groups. | Mention of the perceived injustices felt by evicted communities when describing another study | Trust ecology and the resilience of natural resource management institutions |  |
| 17 | (Young et al. 2016) | Scotland | **community -> agency**  Communities trust in landowners and managers | For agencies to be trustworthy they must show a willingness to share power (Expressed through knowledge of communities having bearing on decision making) | Fairness and justice characterized as communities’ power to influence decisions | The role of trust in the resolution of conservation conflicts |  |
| 18 | (Hamm et al. 2016) | US | **community -> agency**  Land owners trust in NRM institutions | No | Part of their empirical framework is based upon procedural fairness.  Comment that compliance with NRM regulations could lead to lack of autonomy in how land is managed. | On the influence of trust in predicting rural land owner cooperation with natural resource management |  |
| 19 | (Schroeder & Fulton 2017) | US | **community -> agency**  Anglers trust in management decisions | No | Justice as procedure, distribution and ‘voice’ | Voice, Perceived Fairness, Agency Trust, and Acceptance of Management Decisions Among Minnesota Anglers |  |
| 20 | (Coleman & Stern 2018) | US | **community -> agency** | No | Procedural distrust occurs when a process is perceived as unfair | Exploring the Functions of Different Forms of Trust in Collaborative Natural Resource Management |  |
| 21 | (Juerges et al. 2018) | Germany | **bi-directional**  Multiple forest actors perceptions on the importance of trust for conflict resolution | No | Conceptualized as procedural justice | The Role of Trust in Natural Resource Management Conflicts: A Forestry Case Study from Germany |  |
| 22 | (Shirley & Gore 2019) | Brazil | **community -> agency**  Communities trust in scientists and their knowledges | No | Procedural fairness and justice as antecedent to trust | Trust in scientists and rates of noncompliance with a fisheries rule in the Brazilian Pantanal    Trust in scientists and rates of noncompliance with a fisheries rule in the Brazilian Pantanal |  |
| 23 | (Song et al. 2019) | US | **community -> agency**  Civil servants’ perceptions of trust in NRM policy | No | Justice as fairness and procedure | Measuring, mapping and quantifying the effects of trust and informal communication on transboundary collaboration in the Great Lakes fisheries policy network |  |
| 24 | (Wald et al. 2019) | Guam | **community -> agency**  Community trust in scientists, military and environmental  agency | No | Justice as fairness and co-production | The role of trust in public attitudes toward invasive species management on Guam: A case study |  |
| 25 | (Dietsch et al. 2021) | Theoretical | **bi-directional**  Discussion that conservationists elude to the fact that they are stakeholders with certain interests and positions and have dispositions on who they trust or not | Only article in the literature reviewed that draws on power explicitly. They describe power as authority, resource-based power, and legitimacy | Reference made to environmental justice as a term used by liberals that can put off republicans from collaborative engagements. | An understanding of trust, identity, and power can enhance equitable and resilient conservation partnerships and processes. |  |
|  | Total number  discussing the topic |  | 21 advocating for communities trusting in agencies (84%)  4 suggesting bi-directional trust (16%) | 11/25 | 20/25 |  |  |
|  | Reference to a substantive theory |  | **n/a** | 1/25 | 0 |  |  |

**Appendix S2.**

**Search Strategy for focused review**

We were concerned with the role of (dis)trust in conservation and natural resource management (NRM) literatures. Broad search terms were used locate relevant literature, as well as searching the references cited in key texts (Stern & Coleman 2015; Young et al. 2016). To meet the inclusion criteria an article had to concern (dis)trust/trustworthiness in conservation and/or NRM as the main focus study, by either its quantification (qualitative or quantitative) or its theoretical conceptualisation.

**Search strategy of the peer-reviewed literature found in the Web of Science Main Collection Database**

| Topic | Boolean String | Inclusion Criteria | Search focus |
| --- | --- | --- | --- |
| The role of trust in conservation and NRM | (Trust*, distrust, mistrust) AND (Conservation* OR Natural Resource Management) | Discussed or theoretically conceptualised trust as a major component, and/or demonstrated a qualitative, quantitative or mixed method approach for measuring trust.  English language articles | Title, abstract, key words and Key Words Plus |

*Search Results Summary*

The search returned 7231 entries, which were then filtered to 412 articles by removing items from irrelevant disciplines. The remaining 389 abstracts were read or the main text where necessary to assess inclusion according to the above mention criteria. Out of these articles 23 focused on trust as the major component which warranted their inclusion. Two articles were added after the initial literature search.

**Appendix S3**

The connection between justice and trust was initially triggered by analysis of Stern and Coleman’s (2015) typology of trust. In the context of natural resource management, they suggest that trust comprises 1) c*onfidence-based trust*, contingent on past evaluative performances; 2) d*ispositional trust*, which refers to an actor’s pre-determined affinity for trustworthiness; 3) a*ffinitive* trust, shaped by shared values, identities and social connectedness; and 4) s*ystem-based trust or procedural based-trust,* concerning fair procedures and practices. As we were familiar with the tri-dimensional environmental justice framework outlined in the main text, we noticed strong overlap between certain forms of trust and dimensions of justice. For example, procedural trust and procedural justice were an obvious connection. This led us to later explore how this interdependence has a long intellectual tradition and characterises the most prominent relationship between trust and justice (Colquitt & Rodell 2011). Conceptually mapping these different concepts led us to produce an early model for trust and perceived justice (Figure S1) which we have since moved beyond, by adding the concept of trustworthiness and power and moreover now importantly represent the cyclical nature of the interdependence.


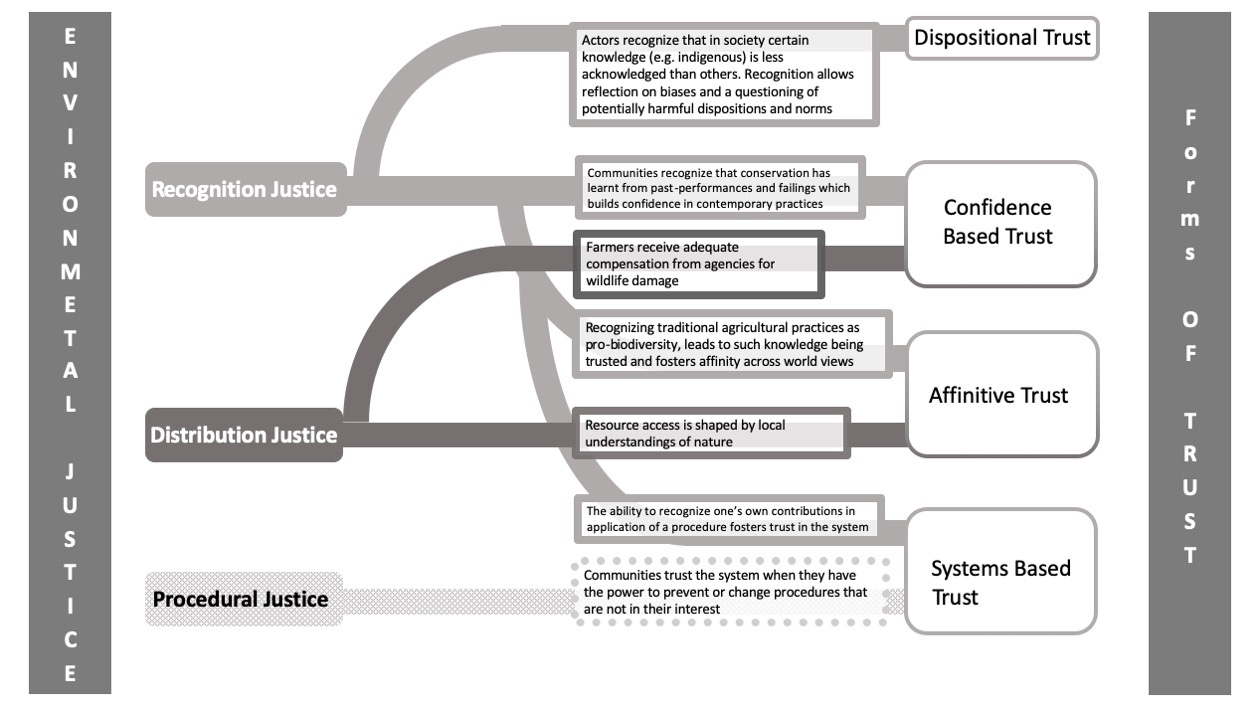


Figure S1 Early attempt at theorizing the interdependence between justice and trust, connecting Stern and Coleman's (2015) typology of trust with three domains of the environmental justice framework.

**Appendix S4**

**The connection between trust and justice in sociology and organisational justice studies**

In our attempt to bridge concepts of trust and justice we reviewed a variety of literatures that attempted to bring these topics together and discuss them below. Whilst they were useful in catalysing our thinking, the contexts were found too distant from conservation settings and ultimately not included in detail in the main text although key citations have been referenced.

Sociology

Scholarship from Sociology holds that without a “social contract there can be no society, and without justice, there can be no social contract” (Emile Durkheim as cited in ((Turowetz & Rawls 2021)). This has been explored in the misunderstandings between marginal and non-marginal people in the workplace. For example, African-American executives routinely deal with white subordinates who question and challenge their trustworthiness by checking with other high authority figures before they follow what their “Black boss asked them to do” (Rawls & Duck 2017). This dispositional distrust by subordinates is a symbol of durable patterns of social injustice, which continues to organize everyday life, compromising relations. Indeed, in this study due to this misrecognition of personhood, the marginalized executives take up defensive behaviors where they withdraw from interactional reciprocity and are then perceived as overly sensitive or non-team players (Rawls & Duck 2017). This suggests there is a cyclical connection between trusting, the functioning of society, where without a just society trusting relationships are compromised.

Organisational Justice

Another area of scholarship which has made explicit efforts towards understanding a justice-trust model is organisational justice studies. The main directionality posited in this field is that perceptions of just treatment lead to building trust (Colquitt & Rodell 2011). For example, fair actions of a supervisor are interpreted as a rewarding action by an employee that engenders reciprocation (Lewicki et al. 2005). Others suggest perceived just actions can increase trust as they are a signifier that if an authority has adhered to fair decision-making previously, they are likely to do so again (Lewicki et al. 2005). This reduces feelings of vulnerability, increasingly a likelihood to trust. In these examples, perceived justice is seen as a currency that fosters social exchange (Colquitt & Rodell 2011). With regards to violations, perceived procedural injustice, such as managers hiring new employees through personal relationships, led existing staff to exhibit lower trust in both employers and the institution (Chen et al. 2004). Overall, the wide literature suggests that “people's trust in other people and organizations grows as a result of fair treatment” (See Lewicki et al. 2005 for a review). There is however, increasing acceptance that the weight often given to perceptions of justice as the primary driver of trust oversimplifies the relationship, and that trust can equally shape perceptions of justice (Colquitt & Rodell 2011). The main theories positing this alternative relational direction are i) the self-interest model where trust is given under anticipation that fairness will be returned (Lewicki et al. 2005), and ii) the group value model where trusting social relations provide the individual with an affirmation and recognition of their identity, leading to perception of self-worth.  Overall the two main directionalities of a trust-justice relationship provide us with two broad views which we take forward for applicability in conservation settings: i) perceptions of justice are a precondition for trust and trustworthiness and, ii) trust and trustworthiness are antecedents for perceptions of justice

Forms of trust and the relation to justice

In organisational justice, it is argued the multi-dimensionality of trust should be given attention as one form of trust being lost will elicit different perceptions of justice than losing another. For example, the expected outcome of trusting someone through a calculus-based rationale is the receipt of a tangible benefit and elicits perceptions of distributional justice.   Relational trust is contrastingly built on commitment to shared values. What is perceived as just is developed according to the peculiarities of the individuals involved, their emotions and desires (Lewicki et al. 2005). Such trust is likely to weather distributional disturbances (Lewicki et al. 2005).  However, it is broken when the other party does something highly unexpected that compromises shared values. Relational trust is therefore more linked to perceptions of misrecognition and procedural injustice through when one party fails to provide adequate explanation for decisions or be a good representative for the others interests (Skarlicki & Folger 1997). However these boundaries are also fluid, forms of trust (the cognitive and relational) cannot necessarily be “neatly separated” as “thought and feeling are always intermingled” (Horowitz 2010),  i.e. a calculative-based trust will arouse emotion if violated. Further environmental justice scholarship shows the interconnectedness of separate domains, one leads into the other (Schlosberg 2009). So for our trust-justice model we suggest trust of any dimension may relate to perceptions of justice in any or multiple domains, but some connections as discussed may be more probable than others.

Attention to forms of trust can also identify how power is constituted in conservation relationships. For example, the character of trust an agency has in a community (e.g. a confidence-based trust to carry out monitoring but not an affinitive-based trust to represent the agencies conservation vision)  can reveal the level of vulnerability organisations wish to accept and key here is the power and control they may not wish to cede (Stevens 2013; Hughes & Vadrot 2019).  Further, someone's or an agency's power comes from being trusted in certain ways. For example a public's pre-determined affinity to trust in conservation, say due to its association with charity, can provide continuing legitimacy and therefore power to such institutions despite violations (Jepson 2005).

**Literature Cited**

Baral N, Stern MJ. 2010. Looking back and looking ahead: local empowerment and governance in the Annapurna Conservation Area, Nepal. Environmental Conservation **37**:54–63.

Chen CC, Chen Y-R, Xin K. 2004. Guanxi Practices and Trust in Management: A Procedural Justice Perspective. Organization ScienceDOI: 10.1287/orsc.1030.0047. INFORMS. Available from https://pubsonline.informs.org/doi/abs/10.1287/orsc.1030.0047 (accessed August 13, 2021).

Coleman K, Stern MJ. 2018. Exploring the Functions of Different Forms of Trust in Collaborative Natural Resource Management. Society and Natural Resources **31**:21–38. Routledge.

Colquitt JA, Rodell JB. 2011. Justice, Trust, and Trustworthiness: A Longitudinal Analysis Integrating Three Theoretical Perspectives. Academy of Management Journal **54**:1183–1206. Academy of Management.

Colquitt JA, Scott BA, LePine JA. 2007. Trust, trustworthiness, and trust propensity: A meta-analytic test of their unique relationships with risk taking and job performance. Journal of Applied Psychology **92**:909–927.

Cvetkovich G, Nakayachi K. 2007. Trust in a High‐concern Risk Controversy: A Comparison of Three Concepts. Journal of Risk Research **10**:223–237. Routledge.

Davenport MA, Leahy JE, Anderson DH, Jakes PJ. 2007. Building Trust in Natural Resource Management Within Local Communities: A Case Study of the Midewin National Tallgrass Prairie. Environmental Management **39**:353–368.

de Vries JR, Aarts N, Lokhorst AM, Beunen R, Munnink JO. 2015. Trust related dynamics in contested land use. Forest Policy and Economics **50**:302–310.

Dietsch AM, Wald DM, Stern MJ, Tully B. 2021. An understanding of trust, identity, and power can enhance equitable and resilient conservation partnerships and processes. Conservation Science and Practice **n/a**:e421.

Ford RM, Williams KJH, Smith EL, Bishop ID. 2014. Beauty, Belief, and Trust: Toward a Model of Psychological Processes in Public Acceptance of Forest Management. Environment and Behavior **46**:476–506.

Gray S, Shwom R, Jordan R. 2012. Understanding Factors That Influence Stakeholder Trust of Natural Resource Science and Institutions. Environmental Management **49**:663–674.

Hamm JA, Hoffman L, Tomkins AJ, Bornstein BH. 2016. On the influence of trust in predicting rural land owner cooperation with natural resource management institutions. Journal of Trust Research **6**:37–62.

Horowitz LS. 2010. “Twenty years is yesterday”: Science, multinational mining, and the political ecology of trust in New Caledonia. Geoforum **41**:617–626.

Hughes H, Vadrot ABM. 2019. Weighting the World: IPBES and the Struggle over Biocultural Diversity. Global Environmental Politics **19**:14–37. MIT Press.

Idrissou L, van Paassen A, Aarts N, Vodouhè S, Leeuwis C. 2013. Trust and hidden conflict in participatory natural resources management: The case of the Pendjari national park (PNP) in Benin. Forest Policy and Economics **27**:65–74.

Jepson P. 2005. Governance and accountability of environmental NGOs. Environmental Science & Policy **8**:515–524.

Juerges N, Viedma A, Leahy J, Newig J. 2018. The Role of Trust in Natural Resource Management Conflicts: A Forestry Case Study from Germany. Forest Science **64**:330–339.

Leahy JE, Anderson DH. 2008. Trust factors in community–water resource management agency relationships. Landscape and Urban Planning **87**:100–107.

Lewicki R, Wiethoff C, Tomlinson E. 2005. What Is the Role of Trust in Organizational Justice? Page in Greenberg J, Colquitt JA, editors. Handbook of Organizational Justice. Taylor & Francis Group.

Metcalf EC, Mohr JJ, Yung L, Metcalf P, Craig D. 2015. The role of trust in restoration success: public engagement and temporal and spatial scale in a complex social-ecological system: Trust in restoration success. Restoration Ecology **23**:315–324.

Rawls AW, Duck W. 2017. “Fractured Reflections” of High-Status Black Male Presentations of Self: Nonrecognition of Identity as a “Tacit” Form of Institutional Racism. Sociological Focus **50**:36–51. Routledge.

Schlosberg D. 2009. Defining environmental justice: theories, movements, and nature. Oxford University Press, Oxford.

Schroeder SA, Fulton DC. 2017. Voice, Perceived Fairness, Agency Trust, and Acceptance of Management Decisions Among Minnesota Anglers. Society & Natural Resources **30**:569–584.

Sharp EA, Thwaites R, Curtis A, Millar J. 2013. Trust and trustworthiness: conceptual distinctions and their implications for natural resources management. Journal of Environmental Planning and Management **56**:1246–1265.

Shirley EA, Gore ML. 2019. Trust in scientists and rates of noncompliance with a fisheries rule in the Brazilian Pantanal. PLOS ONE **14**:e0207973. Public Library of Science.

Skarlicki DP, Folger R. 1997. Retaliation in the workplace: The roles of distributive, procedural, and interactional justice. Journal of Applied Psychology **82**:434–443. American Psychological Association, US.

Smith JW, Leahy JE, Anderson DH, Davenport MA. 2013. Community/Agency Trust and Public Involvement in Resource Planning. Society & Natural Resources **26**:452–471. Taylor & Francis Group.

Song AM, Temby O, Kim D, Saavedra Cisneros A, Hickey GM. 2019. Measuring, mapping and quantifying the effects of trust and informal communication on transboundary collaboration in the Great Lakes fisheries policy network. Global Environmental Change **54**:6–18.

Sponarski CC, Vaske JJ, Bath AJ, Musiani MM. 2014. Salient values, social trust, and attitudes toward wolf management in south-western Alberta, Canada. Environmental Conservation **41**:303–310.

Stern MJ. 2008a. Coercion, voluntary compliance and protest: the role of trust and legitimacy in combating local opposition to protected areas. Environmental Conservation **35**:200–210.

Stern MJ. 2008b. The Power of Trust: Toward a Theory of Local Opposition to Neighboring Protected Areas. Society & Natural Resources **21**:859–875.

Stern MJ, Baird TD. 2015. Trust ecology and the resilience of natural resource management institutions. Ecology and Society **20**:art14.

Stern MJ, Coleman KJ. 2015. The Multidimensionality of Trust: Applications in Collaborative Natural Resource Management. Society & Natural Resources **28**:117–132.

Stevens S. 2013. National Parks and ICCAs in the High Himalayan Region of Nepal: Challenges and Opportunities. Conservation and Society **11**:29.

Turowetz J, Rawls AW. 2021. The development of Garfinkel’s ‘Trust’ argument from 1947 to 1967: Demonstrating how inequality disrupts sense and self-making. Journal of Classical Sociology **21**:3–37. SAGE Publications.

Wald DM, Nelson KA, Gawel AM, Rogers HS. 2019. The role of trust in public attitudes toward invasive species management on Guam: A case study. Journal of Environmental Management **229**:133–144.

Young JC, Searle K, Butler A, Simmons P, Watt AD, Jordan A. 2016. The role of trust in the resolution of conservation conflicts. Biological Conservation **195**:196–202.
